# T-cell polyfunctionality reconstitution after modified anti-thymocyte globulin plus post-transplant cyclophosphamide in allogeneic hematopoietic stem cell transplantation

**DOI:** 10.3389/fimmu.2026.1980998

**Published:** 2026-10-30

**Authors:** Cen Jiang, Luxiang Wang, Lilan Jin, Yanying Gu, Minjing Mao, Yan Miao, Gang Cai

**Affiliations:** 1 Department of Laboratory Medicine, Ruijin Hospital, Shanghai Jiaotong University School of Medicine, Shanghai, China; 2 Shanghai Institute of Hematology, State Key Laboratory of Medical Genomics, National Research Center for Translational Medicine at Shanghai, Ruijin Hospital, Shanghai Jiaotong University School of Medicine, Shanghai, China; 3 Department of Laboratory II, Shanghai Children’s Medical Center, Shanghai Jiaotong University School of Medicine, Shanghai, China

**Keywords:** ATG/PTCy, graft-versus-host disease, hematopoietic stem cell transplantation, immune reconstitution, T-cell function

## Abstract

**Objective:**

Anti-thymocyte globulin combined with post-transplant cyclophosphamide (ATG/PTCy) is increasingly used for graft-versus-host disease (GvHD) prophylaxis; however, functional immune reconstitution after this regimen remains insufficiently characterized. Conventional immune monitoring based on absolute cell counts may not adequately reflect immune competence.

**Methods:**

We retrospectively analyzed 76 patients who received ATG/PTCy (ATG, 2.5 mg/kg/day on days −2 and −1; cyclophosphamide, 50 mg/kg on day +3) following allogeneic peripheral blood stem cell transplantation. Peripheral blood mononuclear cells collected on days +30, +60, +90, +180, and +360 were stimulated with Staphylococcal enterotoxin B (SEB), and T-cell functionality was assessed using COMPASS-derived functionality scores (FS) and polyfunctionality scores (PFS). Associations between immune reconstitution and acute GvHD (aGvHD), chronic GvHD (cGvHD), viral reactivation, and bloodstream infection were assessed.

**Results:**

Within 180 days after transplantation, 34.2% of patients developed aGvHD, including 25.0% with Grade II–IV aGvHD, and 61.8% experienced cytomegalovirus (CMV) reactivation. Peak CMV DNA loads >500 IU/mL were associated with higher incidence of aGvHD, Epstein–Barr virus (EBV) DNA detection, multiple viral infections, and bacteremia. Within 90 days, patients with Grade II–IV aGvHD showed lower CD4+ and CD8+ FS and PFS than those without Grade II–IV aGvHD. Higher CMV DNA levels were associated with impaired T-cell function at days +90 and +180. At day +60, a trend toward an interaction between aGvHD and CMV reactivation were observed for CD8+ T-cell function. At day +180, higher CD8+ T-cell counts and functional activity were associated with increased odds of cGvHD, whereas higher CD4+ T-cell and B-cell levels were associated with lower odds. By day +360, higher B-cell proportions remained inversely associated with cGvHD, whereas CD4+ T-cell levels showed no significant association.

**Conclusions:**

Functional immune monitoring may complement immune cell enumeration and contribute to individualized post-transplant risk assessment in patients receiving ATG/PTCy-based allo-HSCT.

## Introduction

Allogeneic hematopoietic stem cell transplantation (allo-HSCT) remains a cornerstone curative therapy for patients with high-risk hematological malignancies. However, graft-versus-host disease (GvHD) continues to be a major barrier to its success, resulting in substantial morbidity, impaired quality of life, and increased transplant-related mortality (1–3). Anti-thymocyte globulin (ATG) and post-transplant cyclophosphamide (PTCy) are two widely used and effective strategies for GvHD prophylaxis (4, 5). In unrelated donor transplantation, ATG is strongly recommended as part of myeloablative conditioning (MAC) regimens to reduce GvHD risk (6). In haploidentical HSCT, the Chinese “Beijing Protocol”, which incorporates ATG together with unmanipulated granulocyte colony-stimulating factor (G-CSF)-primed bone marrow and peripheral blood stem cells (PBSCs) as the graft source, has achieved outcomes comparable to those of HLA-matched sibling transplantation (7, 8). By contrast, PTCy-based regimens have demonstrated superior efficacy for GvHD prophylaxis in most European and North American haploidentical HSCT centers (5, 9, 10). However, when PBSCs are utilized as the graft source, PTCy alone has been associated with a higher cumulative incidence of GvHD (11, 12). To overcome this limitation, a novel approach combing dose-adjusted ATG with PTCy (ATG/PTCy) has recently been developed to mitigate GvHD risk while preserving the advantages of PBSC-based haploidentical HSCT (5, 13). Emerging evidence indicates that, in both haploidentical and unrelated donor settings, ATG/PTCy reduces the incidence of acute and chronic GvHD (aGvHD and cGvHD), and improves survival compared with ATG alone (14, 15).

Delayed or impaired immune reconstitution after allo-HSCT is a critical determinant of post-transplant morbidity and mortality, increasing the risks of infection and disease relapse. T-cell immune recovery is influenced by multiple factors, including conditioning regimens, thymic function, patient age, graft source, T-cell depletion strategies, donor-recipient HLA disparity, immunosuppressive therapy, and the development of GvHD (3). Although innate immune recovery generally occurs rapidly following transplantation, adaptive immune reconstitution, particularly of the T- and B-cell compartments, is markedly delayed (16, 17). To date, data on immune reconstitution after ATG/PTCy-based haploidentical PBSC transplantation remain scarce. Moreover, most existing studies have primarily focused on quantitative immune recovery, with insufficient assessment of immune functional competence (5, 18, 19). Functional evaluation of immune reconstitution may therefore provide a more informative framework for understanding post-transplant complications and refining prophylactic strategies.

In this present study, we aimed to characterize the kinetics of T-cell functional reconstitution after PBSC transplantation with ATG/PTCy prophylaxis and to explore its association with transplant-related complications.

## Methods

### Patients cohort

This retrospective study included patients aged 18–65 years with hematological malignancies, including acute myeloid leukemia (AML), acute lymphoblastic leukemia (ALL), and myelodysplastic syndromes (MDS), who underwent PBSC transplantation using the combined ATG/PTCy regimen for GvHD prophylaxis (n=76). Patients received either a busulfan-based myeloablative conditioning (MAC, n=56) regimen or reduced-intensity conditioning (RIC, n=20), as previously described (20). Mobilized PBSCs were obtained from haploidentical donors (HIDs, n=65), or HLA-matched unrelated donors (MUDs, n=11). Graft collection and infusion, as well as details of the ATG/PTCy prophylaxis regimen were conducted as previously described (20–22). Briefly, patients received ATG at 2.5 mg/kg/day on days −2 and −1 and cyclophosphamide at 50 mg/kg on day +3. Patients who developed Grade II or higher aGvHD were treated with intravenous methylprednisolone at 2 mg/kg/day. Steroid-resistant aGvHD (SR aGvHD) was defined as disease progression after 3 days or lack of response after 5 days of steroid therapy, and second-line treatment (23).

To ensure cohort homogeneity and minimize confounding, patients underwent bone marrow or umbilical cord blood transplantation, those with contraindications to transplantation (including severe cardiac, renal, or hepatic dysfunction) and those with a history of prior transplantation were excluded. Disease risk was categorized as low, intermediate, or high according to established disease risk indices (22, 23). Overall survival (OS) was defined as the time from transplantation to death from any cause, while event-free survival (EFS) was defined as the time from transplantation to the first occurrence of measurable residual disease (MRD) positivity, relapse, disease progression, or death. Patients who remained alive and event-free were censored at the last follow-up. For nonfatal endpoints, including cytomegalovirus (CMV) reactivation, detection of Epstein-Barr virus (EBV) and human herpesvirus-6 (HHV-6) DNA, and disease relapse, cumulative incidence of relapse (CIR) were estimated, with death before the event of interest treated as a competing event. Patients who experienced neither the event of interest nor the competing event were censored at the last follow-up. Additionally, 12 age- and sex-matched healthy donors underwent T-cell function testing to serve as controls for the patients. The study was approved by the institutional ethics committee (Ethical approval number: RJ-IRB2023130) and conducted in accordance with the Declaration of Helsinki. Written informed consent was obtained from all patients and donors.

### Infection monitoring

All infectious events occurring within ± 4 weeks of blood sampling, including viral, bacterial, and fungal infections diagnosed during routine clinical care, were recorded regardless of severity. No additional microbiological testing was performed beyond standard clinical practice. All patients were classified as high risk for CMV infection according to the 7th edition of the European Conference on Infections in Leukemia (ECIL-7) guidelines (24). CMV surveillance was performed weekly through day +100 and every two weeks thereafter until 12 months post-transplant. Plasma CMV DNA viral load was quantified using a real-time quantitative PCR (qPCR) kit (QIAGEN) with a detection limit of 50 IU/mL. CMV DNAemia was defined as the presence of detectable CMV DNA in plasma. Peak viral load was defined as the highest plasma CMV DNA level measured during the reactivation period. Preemptive therapy was initiated at the treating doctor’s discretion. EBV-DNA and HHV-6-DNA testing was performed concurrently with CMV-DNA monitoring. EBV and HHV-6 DNA positivity was defined as a viral load ≥ 3log10 copies/mL for either virous. When the viral load is below the lower limit of detection, the logarithm of the result is reported as 0. Before Letermovir became available in China, allo-HSCT recipients received preemptive therapy for CMV reactivation (n=39). After its approval, Letermovir was routinely administered as primary CMV prophylaxis (n=37), starting before Letermovir prophylaxis was administered before neutrophil engraftment and continued until day +100 after transplantation, with dose adjustment as needed for concomitant calcineurin inhibitor use. Preemptive therapy was initiated when CMV DNA levels reached ≥1,000 IU/mL in a single measurement or ≥500 IU/mL in two consecutive measurements.

### Immune functional assays and COMPASS analysis

Peripheral blood mononuclear cell (PBMCs) were isolated from heparinized peripheral blood samples by density-gradient centrifugation at each post-transplant time point. PBMCs were plated at 1×10^6^ cells per well in 96-well round-bottom plates and stimulated for 6 hours with Staphylococcal enterotoxin B (SEB, 400ng/mL, Sigma-Aldrich) at 37 °C in 5% CO_2_. PBMCs treated with Dimethylsulfoxide (DMSO, the SEB diluent) alone served as negative controls. During the final 4 hours of stimulation, brefeldin A (BioLegend) was added to a final concentration of 10 µg/mL. A modified intracellular cytokine staining assay was performed as previously reported (25). Production of interleukin (IL)-2, Interferon (IFN)-γ, Tumor necrosis factor (TNF)-α and Macrophage inflammatory protein (MIP)-1β was used to evaluate T-cell functional profiles, allowing identification of 15 functional subsets of CD4+ and CD8+ T cells in response to SEB stimulation. Following labelling with Fixable Viability Dye BV510, cells were stained with an 7-color antibody panel consisting of FITC-anti-CD14 (clone: 63D3), PE-Cy7-anti-CD4 (clone: RPA-T4), APC-Cy7-anti-CD8 (clone: SK1), PerCP-Cy5.5-anti-IL-2 (clone: MQ1-17H12), APC-anti-IFN-γ (clone: 45.B3), Pacific Blue-anti-TNF-α (clone: MAb11), and PE-MIP-1β (clone: D21-1351) (all from Raisecare, Qingdao, China). Samples were analyzed using a BD FACSCantoII flow cytometer (BD Bioscience). FS and PFS were calculated using COMPASS (Combinatorial Polyfunctionality Analysis of Antigen-Specific T-cell Subsets) analysis (26). A minimum threshold of 2000 events was required for both CD4+ and CD8+ T-cell populations (26, 27). Major lymphocyte subsets, including CD3+ T cells, CD4+ and CD8+ T-cell subsets, CD19+ B cells, and CD16+/CD56+ Natural killer (NK) cells, were quantified using the BD Multitest™ 6-Color TBNK Kit (BD Biosciences) with BD Trucount™ Absolute Counting Tubes IVD, following the manufacturer’s instructions. Samples were acquired on a BD FACSCanto II flow cytometer and analyzed using BD FACSCanto Clinical Software v3.0 with the manufacturer’s predefined gating strategy. Detailed assay procedures and population definitions are available in the BD Multitests 6-Color TBNK Kit Technical Data Sheet (Cat. No. 644611).

### Statistical analysis

Except for CMV DNA viral load, which was log10-transformed due to its skewed distribution, continuous variables were analyzed without logarithmic transformation or standardization. Odds ratios (ORs) reflect the change in odds associated with a one-unit increase in continuous variables on their original measurement scale, unless otherwise specified. For clinical interpretability, ORs for selected variables were expressed per clinically meaningful increment, including a 0.1-point increase in CD4+ and CD8+ functional scores (FS and PFS), a 1-log10 increase in CMV DNA viral load, and a 50-cells/μL increase in lymphocyte count. OS and EFS were estimated using the Kaplan-Meier method and compared between groups using the log-rank (Mantel-Cox) test. CIFs were compared between groups using Gray’s test.

Univariable and multivariable logistic regression were performed to examine factors associated with transplant-related complications, including aGvHD within +180 days, CMV reactivation within +100 days, and cGvHD by days +180 and +360. These outcomes were analyzed as binary variables within prespecified clinical assessment windows. Variables with P < 0.2 in univariable analyses were considered for multivariable modeling. The number of model parameters was restricted according to the available outcome events, using the conventional events-per-parameter guideline as a reference. Backward and forward stepwise procedures were used for variable selection. ORs and 95% confidence intervals (CIs) were reported. Given the limited sample size and number of events, these analyses were considered exploratory.

To account for repeated measurements within individual patients, linear mixed-effects models were used to assess longitudinal immune reconstitution. In addition, an individual-level longitudinal analysis was performed to examine the associations of leukocyte and lymphocyte counts with T-cell functional recovery. Further details are provided in the **Supplementary Materials**.

The Bonferroni method was used to adjust multiple comparisons involving immune cell subsets, functional parameters, clinical outcomes, and longitudinal time points. A two-sided Bonferroni-adjusted P value < 0.05 was considered statistically significant. All statistical analyses were performed using R software version 4.3.1 (R Foundation for Statistical Computing, Vienna, Austria).

## Results

### Patients characteristics and clinical outcomes

The demographic characteristics and clinical outcomes are summarized in **Table 1**. Among the 76 patients, the incidence of aGvHD within 180 days post-transplant was 34.2% (26/76), including 25.0% (19/76) with Grade II-IV aGvHD. CMV reactivation occurred in 47 patients (61.8%), while EBV-DNA was detected in 18 patients (23.6%) (**Table 1**). Detection rates for other viral DNA are also shown in **Table 1**. Additionally, 25.0% (19/76) patients developed bloodstream infections within 100 days post-transplant. Within 2 years after transplantation, 16 patients (21.1%) developed moderate-to-severe cGvHD; the CIR and EFS rate were 21.2% and 76.0%, respectively (**Table 1**).

**Table 1:** Baseline characteristics of patients enrolled in this study.

Characteristics	Total (n = 76)	Cohort A (n = 19)	Cohort B (n= 57)	P value
**Median age, years (IQR)**	41.5 (30.8, 54.0)	42 (31, 57)	41.5 (30, 54)	0.737
**Gender, *n* (%)**				0.289
Male	38 (50.0)	7 (36.8)	31 (54.4)	
Female	38 (50.0)	12 (63.2)	26 (45.6)	
**Underlying disease, *n* (%)**				0.133
AML	42 (55.3)	7 (36.8)	35 (61.4)	
ALL	30 (39.5)	10 (52.6)	20 (35.1)	
MDS	3 (4.0)	1 (5.3)	2 (3.5)	
Others	1 (1.3)	1 (5.3)	0 (0)	
**LMV prophylaxis, *n* (%)**	37 (48.7)	8 (42.1)	29 (50.9)	0.691
**Transplantation Procedures, *n* (%)**				1.000
HID	65 (85.5)	17 (89.5)	48 (84.2)	
Unrelated donor	11 (14.5)	2 (10.5)	9 (15.8)	
**HCT-CI scores before allo-HSCT, *n* (%)**				0.358
0 (low risk)	56 (73.7)	13 (68.4)	43 (75.4)	
1–2 (intermediate risk)	17 (22.4)	6 (31.6)	11 (19.3)	
≥ 3 (high risk)	3 (3.9)	0 (0)	3 (5.3)	
**Conditioning, *n* (%)**				0.765
RIC	20 (26.7)	4 (22.2)	16 (28.1)	
MAC	56 (73.3)	15 (77.8)	41 (71.9)	
**RDI, *n* (%)**				0.699
high	30 (39.5)	6 (31.6)	24 (42.1)	
intermediate	38 (50.0)	11 (57.9)	27 (47.4)	
low	8 (10.3)	2 (10.5)	6 (10.5)	
**Blood group disparity, *n* (%)**				0.184
Matched	32 (43.8)	9 (47.4)	23 (40.4)	
Major mismatched	15 (20.6)	6 (31.6)	9 (15.8)	
Minor mismatched	19 (26.0)	4 (21.1)	18 (31.6)	
Major and minor mismatched	7 (9.6)	0 (0)	7 (12.3)	
**CD34^+^ cell counts in graft, median (IQR, ×10^6^/kg)**	8.4 (8.0-10.2)	8.0 (8.0-11.2)	8.3 (7.9-10.2)	0.890
**Median time from HSCT to neutrophil engraftment (IQR, days)**	13 (12-14)	13 (11-14)	13 (12-15)	**0.048**
**Median time from HSCT to platelet engraftment (IQR, days)**	12 (11-14)	11 (10-13)	12 (11-14)	0.099
Acute GvHD in +180 days, *n* (%)				
Grade I - IV	26 (34.2)			
Grade I -IV	19 (25.0)			
Grade II - IV	7 (9.2)			
Chronic GvHD, *n* (%)				
Moderate to severe	16 (21.1)	5 (26.3)	11 (19.3)	0.528
Virus DNAemia in +100 days, *n* (%)				
Herpesviridae				
CMV-DNA, *n* (%)	47 (61.8)	16 (84.2)	31 (54.4)	0.289
Peak load of CMV-DNA (lg IU/mL)	2.5 (0, 3.5)	3.1 (2.5, 3.6)	0 (0, 3.4)	**0.023**
EBV-DNA, *n* (%)	18 (23.6)	5 (26.3)	13 (22.8)	0.765
HHV-6-DNA, *n* (%)	6 (7.9)	2 (10.5)	4 (7.0)	0.636
HSV1/2 -DNA, *n* (%)	5 (6.6)	2 (10.5)	3 (5.3)	0.594
VZV-DNA, *n* (%)	1 (1.3)	0 (0)	1 (1.8)	1.000
Polyomaviridae				
BKV-DNA, *n* (%)	19 (25.0)	8 (42.1)	11 (19.3)	0.067
JCV-DNA, *n* (%)	13 (17.1)	6 (31.6)	7 (12.3)	0.077
**Bloodstream Bacterial infection in +180 days, *n* (%)**	19 (25.0)	4 (21.1)	15 (26.3)	0.766
**2-year EFS (%)**	76.04	72.33	78.15	0.676
**2-year CIR (%)**	21.15	19.95	21.51	0.834

AML, acute myeloid leukemia; ALL, acute lymphocyte leukemia; MDS, myelodysplastic syndrome; LMV: letermovir; HCT-CI, hematopoietic cell transplantation- specific comorbidity index; allo-HSCT, allogeneic hematopoietic stem cell transplantation; MAC, myeloablative conditioning; RIC, reduced intensity conditioning; HLA, human leukocyte antigen; MNC, mononuclear cells; GvHD, graft versus host disease; EBV, Epstein-Barr virus; HSV1, Herpes simplex virus-1; HHV-6, human herpesvirus 6; VZV, varicella-zoster virus; CIR, cumulative incidence of relapse; EFS, event-free survival (event may include disease progression, relapse or death). Bold indicates statistically significant differences (P<0.05).

Patients were then divided into two cohorts according to the occurrence of Grade II-IV aGvHD. Cohort A included patients who developed Grade II–IV aGvHD (n=19), while Cohort B included those who did not develop aGvHD or only developed Grade I aGvHD (n=57). The two cohorts were well balanced with respect to age, gender, underlying disease, blood type disparity, transplantation procedure, graft CD34^+^ cell dose, and refined risk index (RDI). Median time to neutrophil engraftment was shorter in Cohort A than in Cohort B [median (IQR): 13 (11–14) vs 13 (12–15); P = 0.048]. Platelet engraftment also tended to occur earlier in Cohort A, although the difference was not statistically significant [median (IQR): 11 (10–13) vs 12 (11–14); P = 0.099] (**Table 1**). Although the overall rate of CMV reactivation did not differ significantly between two cohorts, the peak CMV-DNA load was much higher in Cohort A than that in Cohort B [median (IQR): 3.1(2.5, 3.6) vs 0(0, 3.4); P = 0.023]. No other clinical outcomes were found significant difference between the two cohorts.

To further analyze the clinical impact of CMV reactivation in patients receiving the ATG/PTCy regimen, we stratified patients according to peak CMV DNA levels using 500 IU/mL as the cutoff value, defining a high-load group (≥500 IU/mL, n = 34) and a low-load group (<500 IU/mL, n = 42). Baseline characteristics and clinical outcomes are summarized in **Supplementary Table 1**. The two groups were comparable with respect to age, gender, underlying disease, blood type disparity, transplantation procedure, graft CD34+ cell dose and RDI, as well as time to neutrophil or platelet engraftment. However, the proportion of patients receiving Letermovir prophylaxis was significantly higher in the low-load group than in the high-load group (73.8% vs 17.6%, P < 0.001) (**Supplementary Table 1**). The incidence of aGvHD, including moderate-to-severe aGvHD, was also markedly increased in the high-load group. Moreover, higher CMV-DNA levels were associated with an increased likelihood of other viral infections or reactivations and bacteremia. However, the incidence of cGvHD was comparable between the two groups (**Supplementary Table 1**). Further multivariable logistic regression analyses showed that Grade II-IV aGvHD was associated with higher odds of CMV reactivation (OR: 2.39, 95% CI: 1.03–5.55; P = 0.046), whereas higher peak CMV DNA viral load was also associated with increased odds of Grade II-IV aGvHD (OR: 1.69; 95% CI: 1.02–2.81; P = 0.044) (**Supplementary Figure 1**).

### Immune reconstitution in HSCT patients with ATG/PTCy regimen

Immune reconstitution were assessed by measuring peripheral blood lymphocyte subsets from 1 to 12 months post-transplant. CD3+ T cells and B cells exhibited progressive recovery in both proportion and absolute counts (**Supplementary Figures 2A, B**). NK cells recovered earliest, with absolute counts remaining relatively stable during the first six months before increasing substantially by one year. Meanwhile, their proportion declined as T- and B-cell populations expanded. CD8+ T cells recovered more rapidly than CD4+ T cells, resulting in inverted CD4+/CD8+ ratios below 1.5 in 84.0%, 83.3%, 88.0%, 93.8%, and 90.9% of patients at day +30, +60, +90, +180, and +360, respectively (**Supplementary Figure 2C**).

T-cell function was evaluated following SEB stimulation (**Supplementary Figure 3**). Based on the expression of IFN-γ, IL-2, TNF-α and MIP-1β, all responsive CD4+ and CD8+ T cells were categorized into 15 distinct functional subsets, including monofunctional (single cytokine producing) and polyfunctional (≥2 cytokines producing) populations. Cytokine-producing subsets gradually increased (**Figure 1A**), transitioning from predominantly monofunctional early post-transplant to increased polyfunctional subsets over time (**Figure 1B**). By one year, SEB-responsive CD4+ and CD8+ T-cell numbers and multifunctional subsets approached healthy control (HC) levels (HCs) (**Figure 1**).

**Figure 1:**
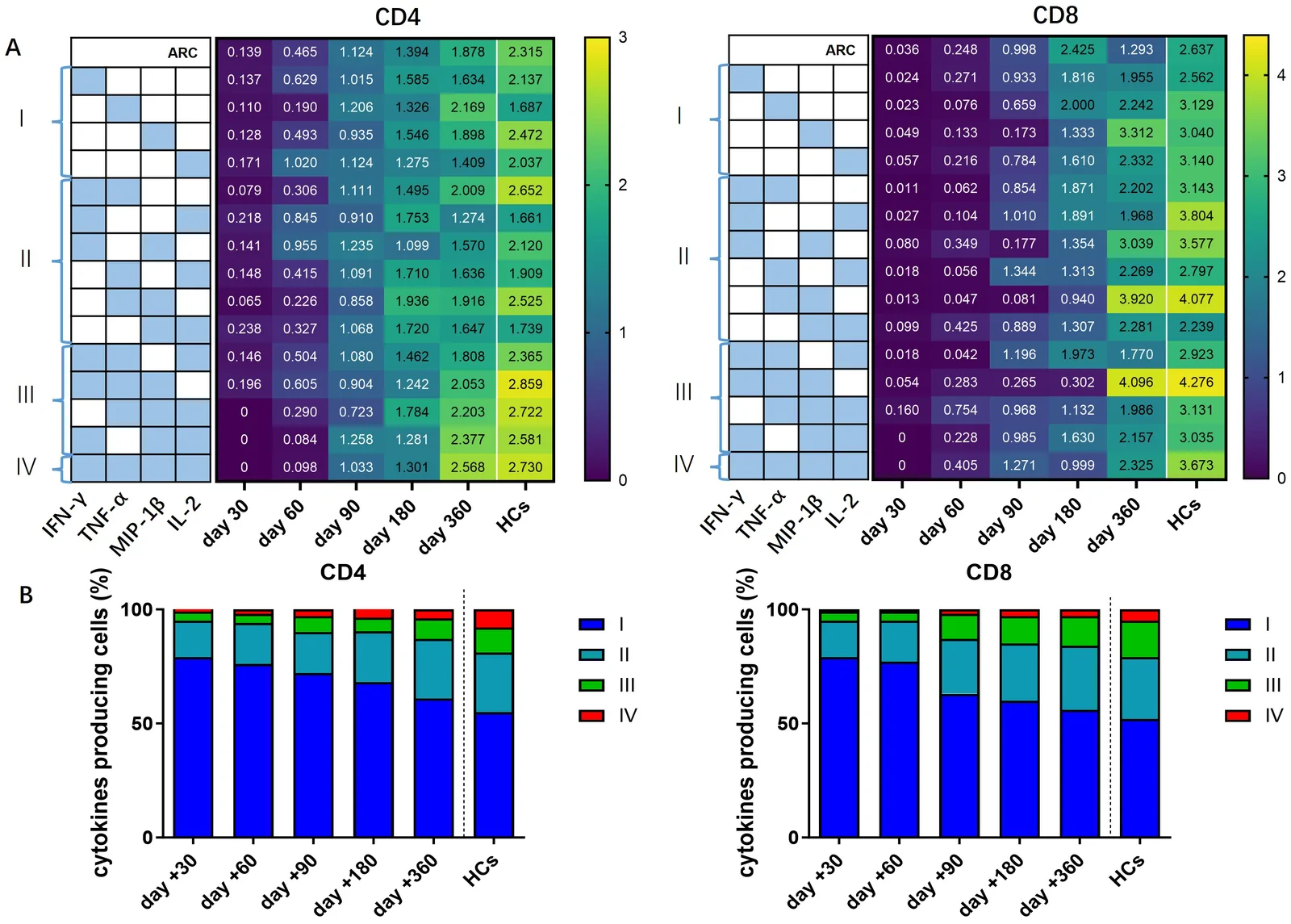
Difference in functional SEB-responsive CD4+ and CD8+ T-cell subsets at multiple post-transplant time points. **(A)** Heatmap showing temporal changes in SEB-stimulated CD4+ (left) and CD8+ (right) T-cell functional subsets in patients and healthy controls (HCs). The underlying data are absolute cell counts (cells/µL), normalized separately for each subset (row). The mean absolute count at each time point (M2) was divided by the mean across all time points (M1) to obtain a dimensionless ratio (M3 = M2/M1). Heatmap values represent normalized ratios relative to the overall mean for each subset. Rows represent individual functional subsets ranked by cytokine expression patterns: I, cells expressing one marker; II, cells expressing two markers; III, cells expressing three markers; and IV, cells expressing all markers. Colored squares indicate the specific cytokines expressed. **(B)** Proportion of cytokine-producing subpopulations within SEB-responsive CD4+ (left) and CD8+ (right) T cell populations in patients at different time points post-transplant and in healthy controls. Columns are composed of four categories: I, cells expressing one marker; II, cells expressing two markers; III, cells expressing three markers; and IV, cells expressing all markers. P values were adjusted for multiple testing using the Bonferroni method. Numbers of cases in each groups: day 30: n=58; day 60: n=60; day 90: n=67; day 180: n=50; day 360: n=50; healthy controls (HCs): n=18.

To further characterize T-cell polyfunctionality, we introduced two metrics: the COMPASS FS and PFS (26). Despite the increase in CD4+ T-cell counts, functional recovery remained delayed. At day +180, more than half of the patients showed low or absent CD4+ T- cell responses (CD4+ FS or PFS < 0.001) (**Supplementary Table 2**), with functional measures approaching HC levels only by one year (**Figure 2**). Conversely, CD8+ T-cell function improved during the first 90 days and remained relatively stable thereafter (**Figure 2**). Although CD8+ FS and PFS showed no significant changes from day +90 onward, both remained below HC levels through day +180. By day +360, 16.0% of patients still exhibited low or absent CD8+ T-cell responses (CD8+ FS or PFS < 0.001) (**Supplementary Table 2**).

**Figure 2:**
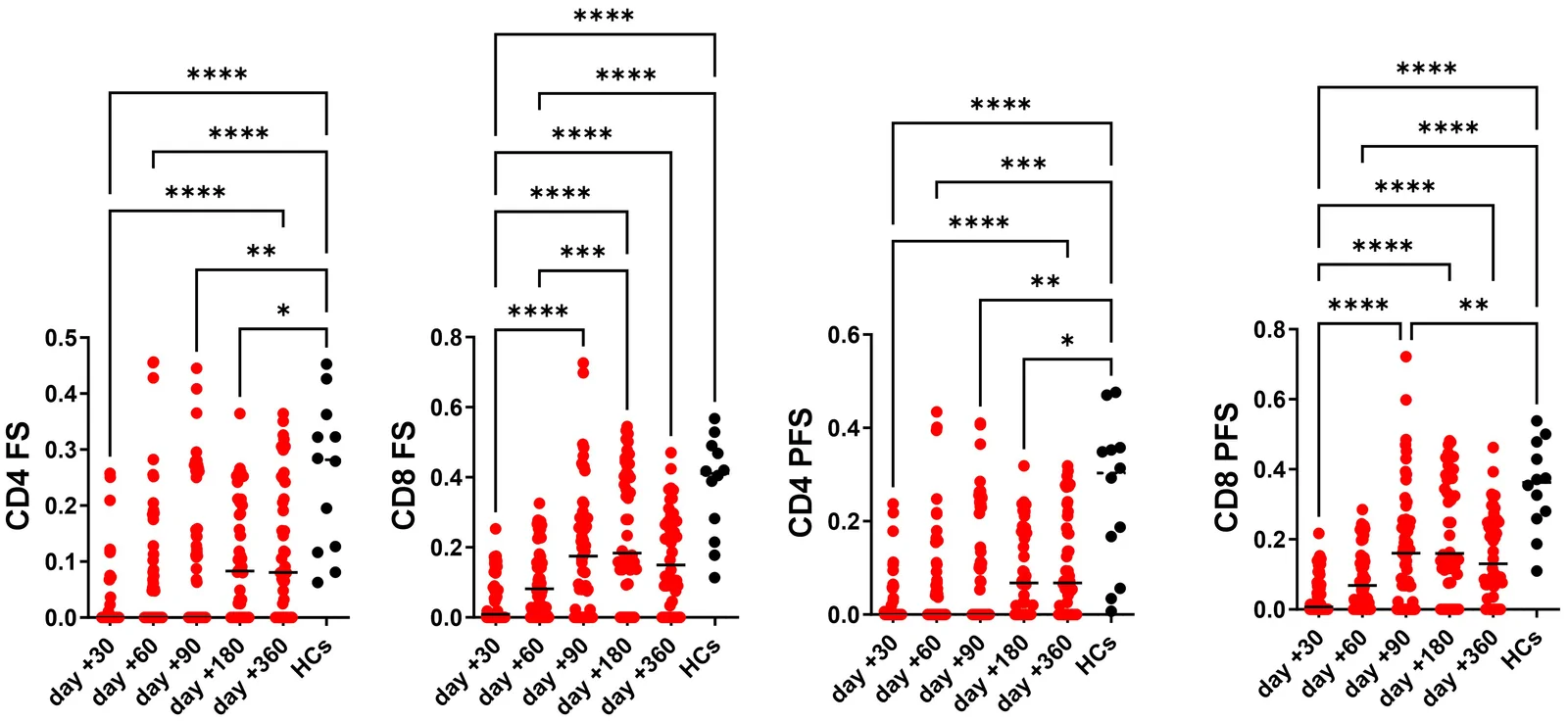
COMPASS-derived functional score (FS) and polyfunctional score (PFS) of CD4+ and CD8+ T cells from patients and healthy controls. Cytokine responses were measured following SEB stimulation. Dot plots depict the distribution of the data, and bar represent median. Comparisons were performed using the one-way ANOVA analysis. Statistically significant P values are shown. *P < 0.05; **P < 0.01; ***P< 0.001 and ****P <0.0001 in pairwise comparisons among the groups. These P values were adjusted for multiple testing using the Bonferroni method. Numbers of cases in each groups: day 30: n=48; day 60: n=60; day 90: n=67; day 180: n=50; day 360: n=50; healthy controls (HCs): n=18.

To further characterize longitudinal immune reconstitution, we examined individual patient trajectories (**Supplementary Figure 4**) and fitted linear mixed-effects models with patient-specific random intercepts and slopes for time (**Supplementary Table 3**). Significant time effects were observed for all nine immune parameters (all P<0.001), including CD3+ (coefficient=6.14), CD4+ (coefficient=1.30), CD8+ T-cells (coefficient=4.63), NK cells (coefficient=1.47), B cells (CD19+ coefficient=0.87), and the four T-cell polyfunctionality measures (CD4+ FS: 0.00078; CD4+ PFS: 0.00073; CD8+ FS: 0.00072; CD8+ PFS: coefficient=0.00066), indicating an overall increase over time (**Supplementary Table 3**). Significant group differences were observed for CD19+ B cells (P = 0.029), CD4+ FS (P<0.001), CD4+ PFS (P<0.001), CD8+ FS (P = 0.002), and CD8+ PFS (P = 0.002), with higher values in Cohort B than in Cohort A. Notably, significant time-by-group interactions were also identified for CD4+ FS (P = 0.002) and CD4+ PFS (P<0.001), indicating greater increases over time in Cohort B. No significant interactions were found for the remaining parameters (all P>0.05) (**Supplementary Table 3**).

We further examined the longitudinal associations of leukocyte and lymphocyte counts with T-cell functional reconstitution. In the within-person centered analysis, both leukocyte and lymphocyte counts showed moderate positive correlations with all four COMPASS-derived functional measures after Bonferroni correction (all P < 0.001; Spearman’s ρ = 0.39–0.44; Pearson’s r = 0.33–0.38) (**Supplementary Table 4**). Similarly, linear mixed-effects models showed significant associations before adjustment for post-transplant time (β = 0.031–0.043, all P < 0.001). However, these associations were attenuated and no longer statistically significant after adjustment for days after transplantation (β = 0.007–0.013, all P > 0.10) (**Supplementary Table 5**). These findings suggest that the observed correlations largely reflect shared temporal patterns of immune recovery rather than associations independent of time since transplantation.

### aGvHD and CMV reactivation were associated with delayed functional T-cell immune reconstitution

Although Grade II-IV aGvHD did not significantly affect the recovery of T-cell counts, it was closely associated with impaired functional T-cell reconstitution. Within the first 90 days after transplantation, CD3+, CD4+ and CD8+ T-cell counts did not differ significantly between Cohorts A and B. However, COMPASS-derived FS and PFS values for both CD4+ and CD8+ T cells were significantly lower in Cohort A than in Cohort B. At day +30, no significant difference in CD4+ FS or PFS was observed between the two cohorts (**Figure 3**), likely because CD4+ T-cell counts were very low in both groups during the early post-transplant period. Furthermore, B-cell levels at day +60 and +90 were significantly lower in patients with Grade II-IV aGvHD than in the control group (**Figure 3**). By days +180 and +360, T-cell function had converged to similar levels in the two cohorts (**Figure 3**).

**Figure 3:**
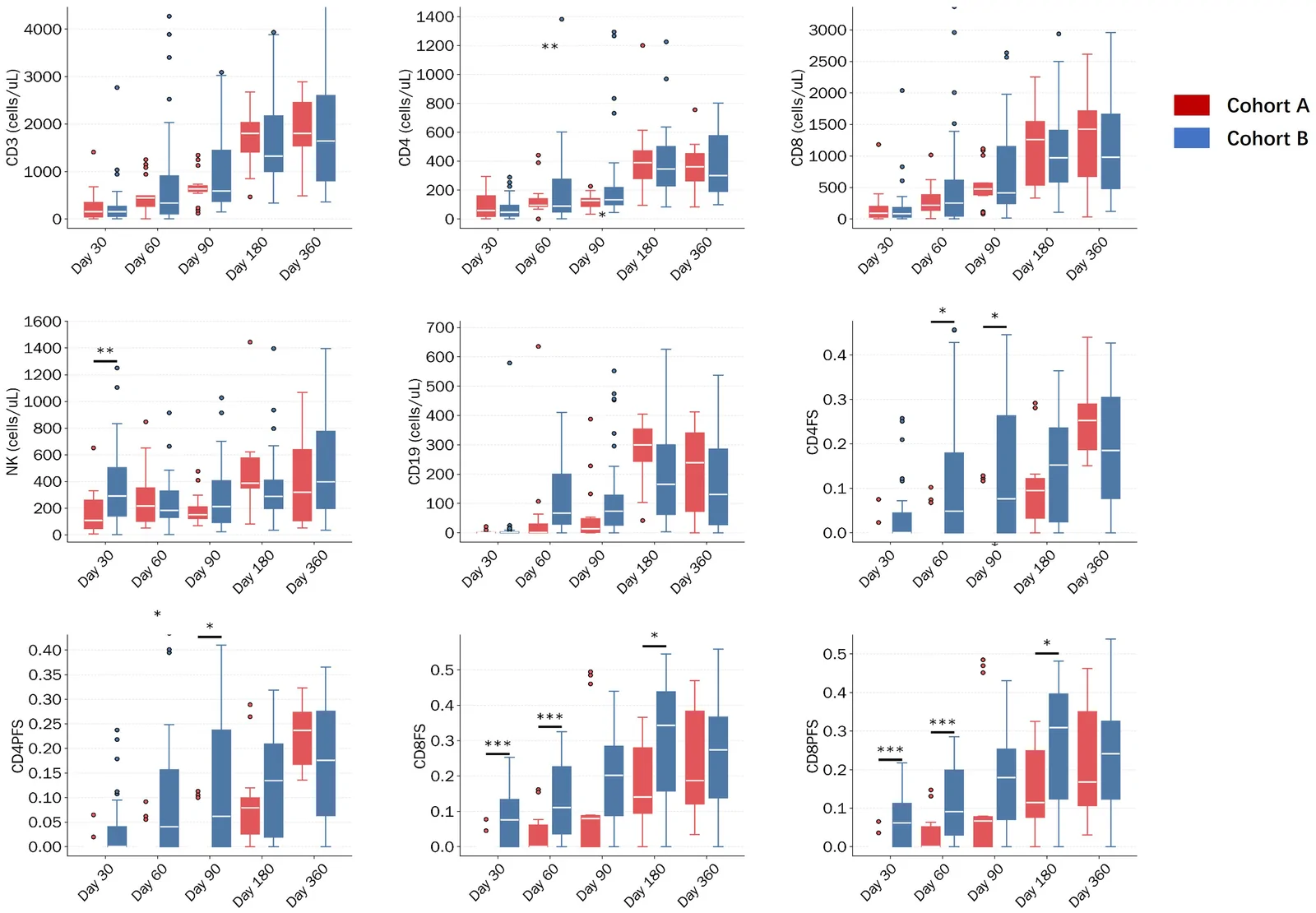
Longitudinal recovery of lymphocyte subsets (CD3+, CD4+, CD8+, NK, and CD19+ cells) and T-cell polyfunctionality (CD4+FS, CD4+PFS, CD8+FS, and CD8+PFS) in HSCT patients with Grade II–IV aGVHD (Cohort A) and those with Grade 0–I aGVHD (Cohort B). Between-group comparisons were performed using nonparametric rank-sum tests, with Bonferroni correction for multiple comparisons. Statistically significant differences are indicated as follows: *P < 0.05, **P < 0.01, and ***P < 0.001 (Bonferroni-adjusted). The numbers of patients in Cohorts A and B at each time point were 18 and 40 at Day +30, 17 and 43 at Day +60, 15 and 52 at Day +90, 12 and 38 at Day +180, and 10 and 40 at Day +360, respectively.

We also analyzed the impact of CMV reactivation on functional T-cell reconstitution. Although CMV reactivation appeared to be associated with T-cell expansion, particularly CD8+ T-cell expansion, no significant differences in CD4+ or CD8+ T-cell function were observed between the high-load and low-load groups. However, by day +90 and day +180, when T-cell counts had reached equilibrium in both groups, T-cell function was significantly lower in the high-load group than in the low-load group (**Figure 4**). By one year after transplantation, T-cell function in the high-load group had recovered to levels comparable to those in the low-load group (**Figure 4**).

**Figure 4:**
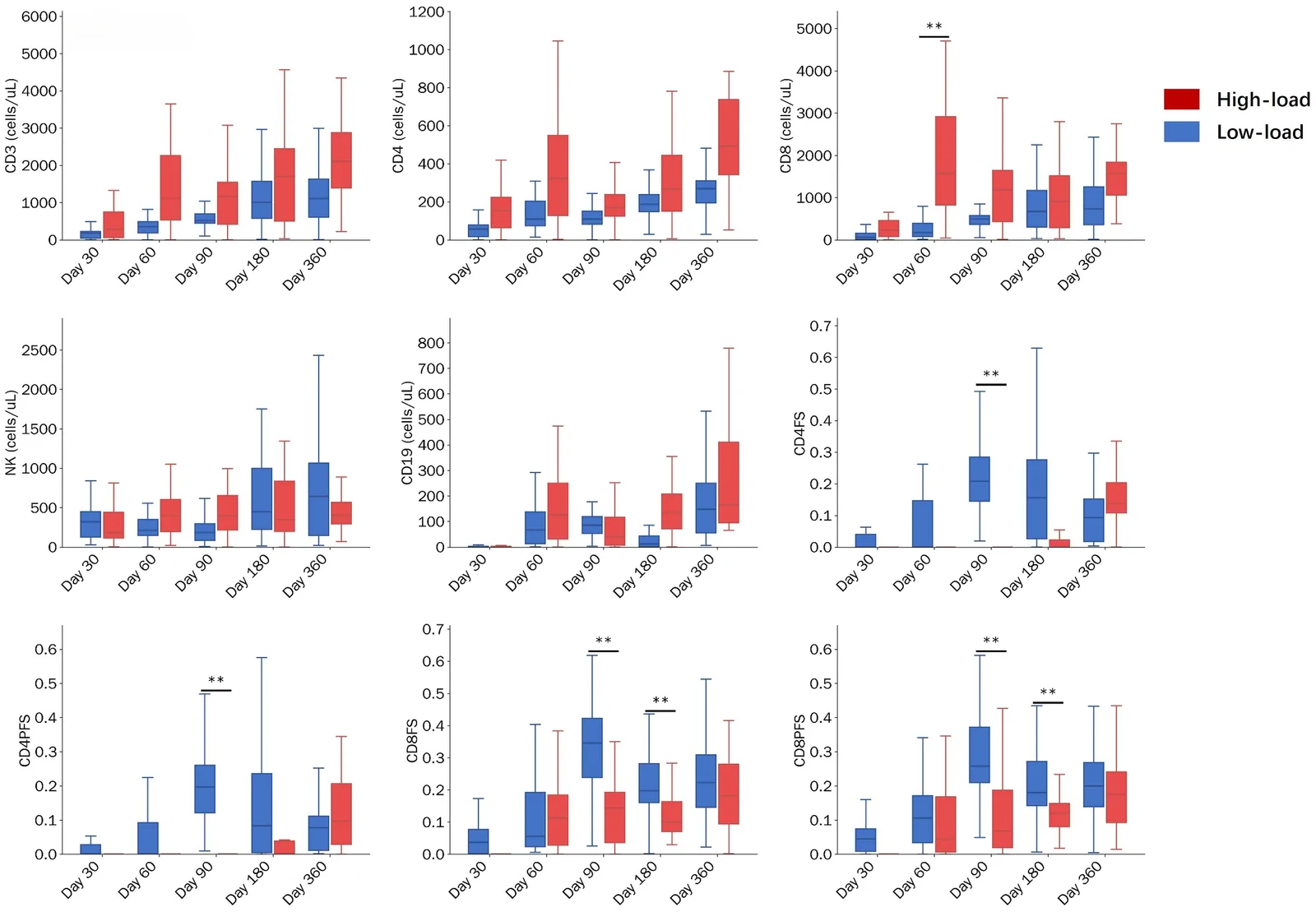
Longitudinal recovery of lymphocyte subsets (CD3+, CD4+, CD8+, NK, and CD19+ cells) and T-cell polyfunctionality (CD4+FS, CD4+PFS, CD8+FS, and CD8+PFS) in HSCT patients stratified into high- and low-CMV DNA groups. Between-group comparisons were performed using nonparametric rank-sum tests, with Bonferroni correction for multiple comparisons. Statistically significant differences are indicated as follows: *P < 0.05 and **P < 0.01 (Bonferroni-adjusted). The numbers of patients in high-load and low-load at each time point were 18 and 40 at Day +30, 27 and 33 at Day +60, 29 and 38 at Day +90, 16 and 34 at Day +180, and 18 and 32 at Day +360, respectively.

Two-way analysis of variance (ANOVA) was further performed to examine the interaction between aGVHD and CMV DNA load in relation to T-cell function. At day +60, a trend toward an interaction between aGvHD and CMV reactivation was observed for CD8+ T-cell function (FS: F value = 4.0536, P = 0.0516; PFS: F value = 4.2182, P = 0.0437) (**Supplementary Figure 5**). Specifically, among patients with no or Grade I aGvHD, CD8+ T-cell polyfunctionality was significantly lower in high-load group than in the low-load group. In contrast, the difference was less pronounced among patients with Grade II-IV aGvHD, suggesting that the association between CMV DNA load and CD8+ T-cell polyfunctionality may vary with aGvHD severity. No significant interactions were observed at other time points or for other T-cell functional parameters (**Supplementary Figure 5**).

### Association of T-cell functionality with cGvHD

To identify risk factors for cGvHD in patients receiving ATG/PTCy prophylaxis, we analyzed the impact of T-cell functional indices and early post-transplant events on cGvHD. Due to the small number of patients with moderate-to-severe cGvHD in the day +30, day +60, and day +90 cohorts, it was difficult to obtain robust data. At day +180, univariable logistic regression analysis revealed that higher proportion and greater function activity of CD8+ T cells, including CD8+ FS and PFS, were associated with an increased risk of moderate-to-severe cGvHD. Conversely, higher levels of CD4+ T cells and B cells were associated with a lower risk of moderate-to-severe cGvHD (**Supplementary Table 6**). Multivariable logistic regression analysis largely confirmed these findings, although CD8+ PFS did not reach statistical significance in the multivariable model (**Figure 5A**). At day +360, univariable analysis demonstrated that a higher CD8+ T-cell proportion was associated with increased odds of cGvHD (OR 1.87, P = 0.013), whereas a higher CD19+ B-cell proportion was associated with lower odds of cGvHD (OR 0.44, P = 0.0002). No significant associations were observed for CD4+ T-cell percentage or T-cell functional scores (**Supplementary Table 7**). Furthermore, the occurrence of Grade II-IV aGvHD was also associated with an increased risk of cGvHD (**Figure 5B**). Notably, bacteremia during the early post-transplant period (within 100 days) was associated with a reduced risk of cGvHD (**Figures 5A, B**).

**Figure 5:**
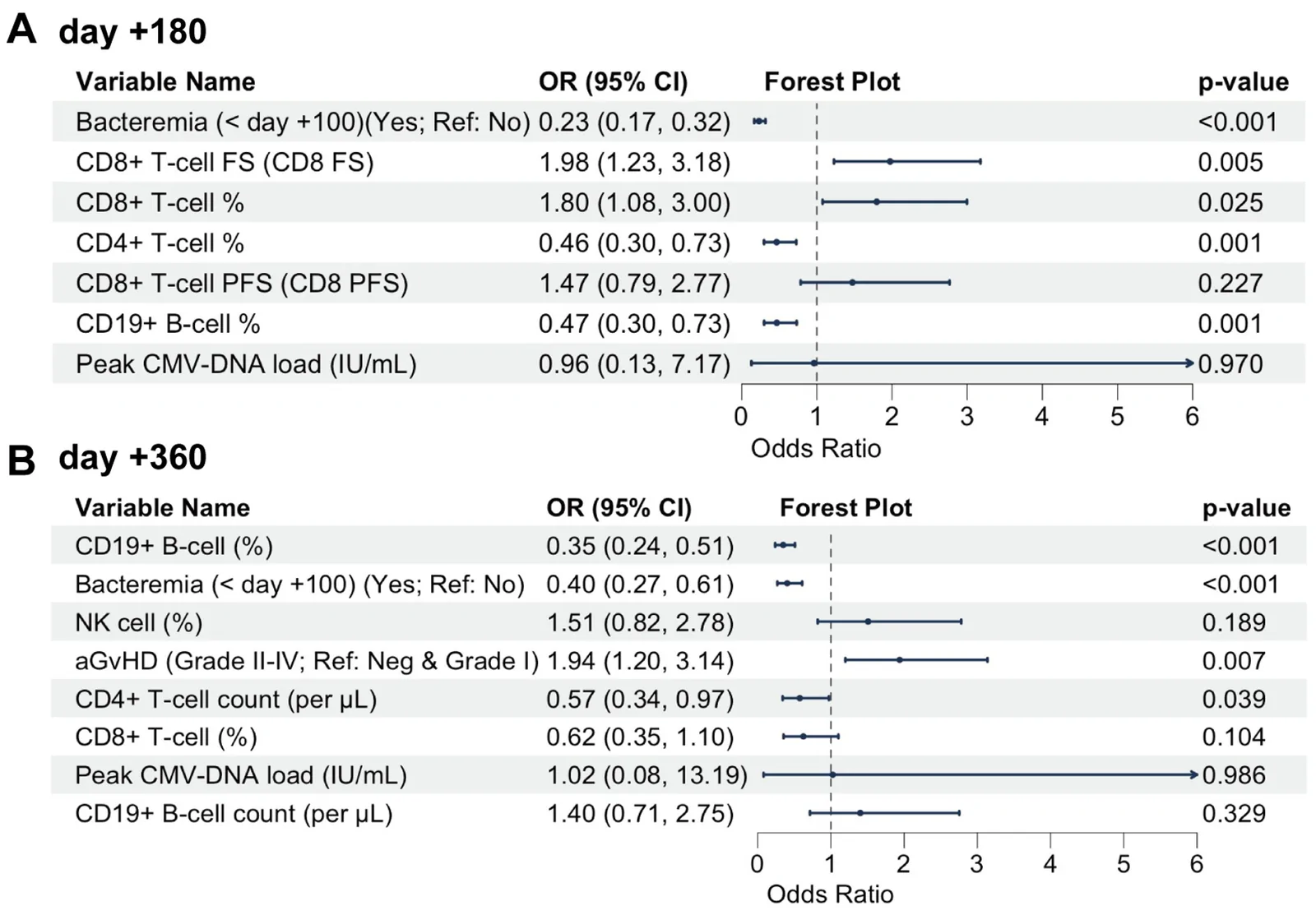
Multivariable logistic regression analysis of clinical and immune parameters associated with chronic graft-versus-host disease (cGVHD) on day +180 **(A)** and day +360 **(B)**. Candidate predictors were assessed using univariable logistic regression (**Supplementary Tables 6**, **7**). Variables with P < 0.20 were entered into multivariable models, followed by backward stepwise selection based on the Akaike information criterion (AIC). For the Day +180 model, 50 patients were analyzed, including 15 with cGVHD. Of the 17 variables screened, 7 met the P < 0.20 criterion and were entered into the multivariable model. For the Day +360 model, 50 patients were analyzed, including 13 with cGVHD. Of the 17 variables screened, 8 met the screening criterion and were entered into the multivariable model. Predictors retained in the final models are shown as odds ratios (ORs; points) with 95% confidence intervals (CIs; horizontal lines). The vertical dashed line at OR = 1 indicates no association; values above or below 1 indicate higher or lower odds of cGVHD, respectively. All reported P values were Bonferroni-adjusted for multiple comparisons. FS, functional score; PFS, polyfunctional score; CMV, cytomegalovirus; OR, odds ratio; CI, confidence interval.

## Discussion

Accumulating evidence has established the clinical efficacy of ATG/PTCy regimen in reducing GvHD without compromising the therapeutic benefits of haploidentical PBSC transplantation (5, 13, 21). However, data on immune reconstitution under this combined immunosuppressive strategy remain limited, particularly from a functional immunological perspective. In this retrospective study, we analyzed clinical outcomes following ATG/PTCy-based allogeneic PBSC transplantation, predominantly involving haploidentical and matched unrelated donors, and characterized longitudinal immune function recovery by assessing peripheral blood mononuclear cell responses to SEB during the first-year post-transplant.

Firstly, we evaluated clinical outcomes of patients receiving ATG/PTCy. Consistent with previous real-world studies, the incidence of Grades II–IV aGVHD in our cohort was within the range reported in the literature (5, 6, 28, 29). No significant differences were observed between patients who developed Grade II-IV aGvHD and those who did not with respect to age, sex, underlying disease, transplantation procedure, conditioning regimen, hematopoietic cell transplantation-specific comorbidity index (HCT-CI) score, donor-recipient blood type matching, or graft stem cell dose. However, patients who developed Grade II-IV aGvHD achieved granulocyte engraftment significantly earlier than those who did not. Furthermore, patients with aGvHD also achieved platelet engraftment also tended to occur earlier in patients with aGvHD, although the difference did not reach statistical significance (**Table 1**). These findings may suggest more rapid graft engraftment is associated with an increased risk of aGvHD. Multiple studies have shown that aGvHD and CMV reactivation are mutually reinforcing risk factors (30–32), and our results fully support this conclusion (**Supplementary Figure 1**). Patients with Grade II-IV aGvHD had significantly higher CMV DNA loads than their controls. Because our cohort included patients who received either a preemptive treatment strategy or letermovir prophylaxis, the cumulative incidence of CMV reactivation within 100 days post-transplant was 61.8% (47/76). Using a cutoff value of 500 IU/mL, the high-load group exhibited significantly higher incidences of both overall aGvHD and Grade II-IV aGvHD than the low-load group. In addition, the high-load group had significantly higher rates of EBV DNA detection, multiple viral infections, and bacteremia than the low-load group. Together, these findings further support the detrimental effect of CMV reactivation on post-transplant immune reconstitution.

Immune reconstitution is a key determinant of transplant outcomes; however, whether numerical recovery of immune cells after transplantation truly reflects restoration of immune function remains controversial. In this longitudinal analysis of 76 HSCT recipients, leukocyte and lymphocyte counts showed moderate within-person correlations with T-cell functional recovery (ρ=0.39–0.44) (**Supplementary Table 4**). However, these associations were no longer significant after adjustment for time since transplantation, suggesting that they largely reflected parallel recovery trajectories rather than independent changes in T-cell function (**Supplementary Table 5**). These findings suggest that conventional blood counts may not fully capture functional immune reconstitution, highlighting the potential value of direct functional immune monitoring.

However, reliable methods for assessing immune function in transplant recipients remain limited, particularly for evaluating adaptive T-cell responses. The traditional use of IFN-γ expression as a surrogate for T-cell reactivity has important limitations, as it does not fully capture the breadth and quality of T-cell responses (33). A more informative approach is to assess multiple activation markers simultaneously or to evaluate T-cell multifunctionality, although standardization of such measurements remains a challenging. In this study, to systematically evaluate overall T-cell responsiveness, the superantigen SEB was used to stimulate T cells, and their capacity to produce multiple cytokines (IFN-γ, TNF-α, IL-2 and MIP-1β) was assessed. T cells capable of producing two or more cytokines were defined as polyfunctional T cells. According to these cytokine expression profiles, SEB-responsive T cells were classified into 15 functional subsets, 11 of which were polyfunctional (**Figure 1A**). In most but not all samples, IFN-γ was the dominant cytokine secreted after SEB stimulation. Over time after transplantation, however, both CD4+ and CD8+ T cells exhibited a gradual increase in their ability to produce various cytokines (**Figure 1A**), accompanied by an increase in the proportion of multifunctional cells (**Figure 1B**). By integrating information across multiple cytokines profiles, COMPASS provides a more comprehensive representation of T-cell functional quality than single-parameter assays (34, 35). The FS and PFS derived from the COMPASS also provide a more intuitive framework for characterizing recovery of CD4+ and CD8+ T-cell function. The immune function of CD8+ T cells showed a gradual recovery within 90 days post-transplant, but stabilized thereafter. The recovery of CD4+ T-cell function lagged behind that of CD8+ T-cells; their responsiveness to SEB remained at low levels for the first six months post-transplant and did not show a rapid upward trend until +180 days post-transplant (**Figure 2**). These findings suggest that, during the early post-transplant period, adaptive immune protection may rely primarily on CD8+ T cells, while the full restoration of adaptive immune capacity likely depends on subsequent recovery of CD4+ T-cell function.

We followed this cohort for at least one year to monitor the immune reconstitution process and analyzed the relationship between post-transplant complications (particularly aGvHD and CMV reactivation) and immune recovery. During the first six months after transplantation, neither aGvHD nor CMV reactivation appeared to be significantly associated with quantitative T-cell recovery; however, patients who developed either complication exhibited a marked delay in functional T-cell reconstitution. Patients who developed aGvHD showed significantly lower T-cell polyfunctionality than those without aGvHD within the first 90 days post-transplant (**Figure 3**). Because aGvHD predominantly occurs within the first 100 days post-transplant and more severe cases are treated with immunosuppressive corticosteroids, the impaired T-cell polyfunctionality observed in these patients may also have been influenced by immunosuppressive therapy. In contrast, the effect of CMV reactivation on T-cell function was primarily evident between +90 and +180 days (**Figure 4**). Historically, CMV reactivation has been concentrated in the early post-transplant period (36); however, the introduction of letermovir prophylaxis has significantly delayed its onset in many patients (37). These distinct temporal patterns of impaired T-cell polyfunctionality may therefore reflect the timing of different post-transplant complications. Using two-way analysis of variance, we found a trend toward an interaction between aGvHD and CMV reactivation on CD8+ T-cell function only at day +60. This finding suggests that the first two months post-transplant may be a particularly vulnerable period, during which prevention and close monitoring of various complications are especially important.

Chronic GvHD is another common complication following transplantation (38). In our cohort, higher proportions of CD19+ B cells were associated with a lower likelihood of cGvHD, whereas higher CD8+ T-cell percentages and functional activity were associated with increased odds at day +180 (**Figure 5A**). Notably, higher CD4+ T-cell proportions were associated with lower odds of cGvHD at day +180, but this association was not statistically significant at day +360. These findings may reflect the involvement of persistent CD8+ T-cell activation in tissue injury, while B-cell recovery may indicate a more balanced immune reconstitution. However, because day +180 overlaps with the typical onset period of cGVHD, increased CD8+ T-cell activity may also reflect subclinical or established disease rather than precede its development. Earlier longitudinal sampling will be required to clarify this temporal relationship. The inverse association between early bacteremia and cGvHD should also be interpreted cautiously (**Figure 5**).

This study has several limitations, including its retrospective design, relatively small sample size, and limited number of Grade II-IV aGvHD and moderate-to-severe cGvHD events. These factors preclude causal inference and may increase the risk of overfitting in multivariable analyses. The limited number of events for NRM, OS, and CIR precluded reliable multivariable survival analyses incorporating time-dependent immune reconstitution parameters. In addition, although logistic regression was used to assess outcomes within predefined clinical time windows, it did not account for event timing or competing risks. Therefore, the observed associations should be interpreted cautiously. Larger prospective multicenter studies with sufficient outcome events and appropriate time-to-event models, including Cox regression and competing-risk approaches, are needed to validate the prognostic value of T-cell functional parameters.

In summary, we characterized longitudinal immune reconstitution during the first year after ATG/PTCy-based allo-HSCT in a predominantly haploidentical cohort, with particular emphasis on T-cell function. The immune reconstitution pattern observed in our cohort, characterized by relatively rapid CD8+ T-cell functional recovery within the first six months and delayed CD4+ T-cell functional recovery may reflect the intensive *in vivo* T-cell depletion associated with the combined ATG/PTCy regimen. Compared with haploidentical transplantation using PTCy alone (39, 40), the combined regimen may lead to more pronounced early immunosuppression, particularly impaired CD4+ T-cell function, while potentially providing better GvHD control without sustained impairment of functional recovery at one year. Similarly, compared with the ATG-based “Beijing Protocol” (39, 40), ATG/PTCy appears to be associated with delayed CD4+ T-cell recovery but potentially lower rates of cGVHD. In contrast, matched sibling donor (MSD) transplantation, which generally involves less intensive T-cell depletion, has been associated with more rapid CD4+ T-cell recovery (41). The prolonged CD4+ functional impairment observed in our predominantly haploidentical cohort may therefore reflect differences in donor type and GvHD prophylaxis. These findings suggest a potentially extended period of vulnerability to opportunistic infections during the first six months after transplantation, highlighting the importance of infection prevention and functional immune monitoring in patients receiving ATG/PTCy. In addition, we believe that the functional parameters derived from the COMPASS assay reveal an association between T-cell multifunctionality and major post-transplant complications, including aGvHD, cGvHd, and CMVreactivation.

Therefore, functional immune monitoring may complement conventional immune cell counts in identifying patients with delayed immune reconstitution who may benefit from closer surveillance and individualized post-transplant management.

## Data Availability

The raw data supporting the conclusions of this article will be made available by the authors, without undue reservation.
