# Supplementary material for: T-cell polyfunctionality reconstitution after modified anti-thymocyte globulin plus post-transplant cyclophosphamide in allogeneic hematopoietic stem cell transplantation
Source: Front Immunol. 2026 Oct 30;17:1980998. doi: 10.3389/fimmu.2026.1980998 (PMC13759409; doi:10.3389/fimmu.2026.1980998)
Supplement: Supplementary file 1 [file SupplementaryFile1.docx]

**Supplemental Methods**

**Association Between Immune Reconstitution and Routine Blood Parameters**

**Statistical analysis**

To examine the longitudinal associations between routine blood counts and T-cell functional reconstitution, we analyzed 285 paired observations from 76 patients at Days +30, +60, +90, +180, and +360 after transplantation. The laboratory parameters included white blood cell (WBC) and lymphocyte counts, while T-cell function was assessed using COMPASS-derived functionality scores (FS) and polyfunctionality scores (PFS) for CD4+ and CD8+ T cells.

The primary analysis used within-person centering to distinguish intra-individual changes from between-patient differences. For each patient, the mean of each variable across available time points was calculated, and within-person deviations were obtained by subtracting the patient-specific mean from each observation. Spearman rank correlation coefficients (ρ) were then calculated between the centered blood counts and T-cell functional scores. Pearson correlation coefficients were also calculated as a sensitivity analysis.

Bootstrap resampling (2,000 iterations) was used to estimate 95% confidence intervals for the correlation coefficients. Bonferroni correction was applied to the eight primary comparisons (two laboratory parameters × four functional measures), with statistical significance defined as an unadjusted P value < 0.00625 (0.05/8).

Linear mixed-effects models with patient-specific random intercepts were further fitted to assess these associations. The initial models included within-person deviations in blood counts as predictors of T-cell functional scores. Additional models adjusted for days after transplantation as a continuous covariate to examine whether the associations persisted after accounting for time-related immune recovery. All analyses were performed using Python version 3.12.

**Supplemental Table 1. Comparison of patients with high and low CMV DNA loads**

| **Characteristics** | **≥ 500 IU/mL**  **(n = 34)** | **< 500 IU/mL**  **( n = 42)** | **P value** |
| --- | --- | --- | --- |
| **Median age, years (IQR)** | 41.5(34, 53) | 44 (30.3, 54) | 0.950 |
| **Gender, *n* (%)** |  |  | 0.818 |
| Male | 18 (52.9) | 20 (47.6) |  |
| Female | 16 (47.1) | 22 (52.4) |  |
| **Underlying disease, *n* (%)** |  |  | 0.769 |
| AML | 20 (58.8) | 22 (52.4) |  |
| ALL | 13 (38.2) | 17 (40.5) |  |
| MDS | 1 (2.9) | 2 (4.8) |  |
| Others | 0 (0) | 1 (2.4) |  |
| **LMV prophylaxis, *n* (%)** | 6 (17.6) | 31(73.8) | **0.000** |
| **Transplantation Procedures, *n* (%)** |  |  | 0.967 |
| Haploidentical donor | 29 (85.3) | 36 (85.7) |  |
| Unrelated donor | 5 (14.7) | 6 (14.3) |  |
| **HCT-CI scores before allo-HSCT, *n* (%)** |  |  | 0.248 |
| 0 (low risk) | 27(79.4) | 29(69.0) |  |
| 1–2 (intermediate risk) | 7(20.6) | 10(23.8) |  |
| ≥ 3 (high risk) | 0(0) | 3(7.2) |  |
| **Conditioning, *n* (%)** |  |  | 0.610 |
| RIC | 24(70.6) | 30(76.2) |  |
| MAC | 10(29.4) | 10(23.8) |  |
| **RDI, *n* (%)** |  |  | 0.521 |
| high | 11(32.4) | 19(45.2) |  |
| intermediate | 19(55.9) | 19(45.2) |  |
| low | 4(11.8) | 4(9.5) |  |
| **Blood group disparity, *n* (%)** |  |  | 0.538 |
| Matched | 16(47.1) | 16(38.1) |  |
| Major mismatched | 8(23.5) | 7(16.7) |  |
| Minor mismatched | 8(23.5) | 14(33.3) |  |
| Major and minor mismatched | 2(5.9) | 5(11.9) |  |
| **CD34^+^ cell counts in graft, median (IQR, ×10^6^/kg)** | 8.7(7.7-11.2) | 8.4(8.0-10.1) | 0.766 |
| **Median time from HSCT to neutrophil engraftment (IQR, days)** | 13(12-14) | 13(12-14.75) | 0.225 |
| **Median time from HSCT to platelet engraftment (IQR, days)** | 11(11-13) | 12(11-14) | 0.290 |
| **Acute GvHD in +180 days, *n* (%)** |  |  |  |
| Grade I - IV | 17(50.0) | 8(21.4) | **0.007** |
| Grade ⅠI -IV | 13(38.2) | 6(14.3) | **0.031** |
| Grade ⅡI - Ⅳ | 6(17.6) | 1(2.4) | **0.041** |
| **Chronic GvHD, *n* (%)** |  |  |  |
| Moderate to severe | 9(26.5) | 7(16.7) | 0.398 |
| **Other virus detection** |  |  |  |
| EBV-DNA, *n* (%) | 13(38.2) | 5(11.9) | **0.013** |
| Multiple viral infection*, *n* (%) | 28(82.4) | 12(28.6) | **0.000** |
| **Bloodstream Bacterial infection in +180 days, *n* (%)** | 31(91.2) | 26(61.9) | **0.004** |

Abbreviations: AML, acute myeloid leukemia; ALL, acute lymphocyte leukemia; MDS, myelodysplastic syndrome; LMV: letermovir; HCT-CI, hematopoietic cell transplantation- specific comorbidity index; allo-HSCT, allogeneic hematopoietic stem cell transplantation; MAC, myeloablative conditioning; RIC, reduced intensity conditioning; GvHD, graft versus host disease; EBV, Epstein-Barr virus;

* the virus in multiple viral infection including HSV-1/2, HHV-6, VZV, BKV and JCV.

**Supplemental Table 2. Proportion of patients with low or absent T-cell responses to SEB stimulation.**

| CD4 FS | < 0.001 | < 0.001 | ≥ 0.001 | ≥ 0.001 |
| --- | --- | --- | --- | --- |
| CD8 FS | < 0.001 | ≥ 0.001 | < 0.001 | ≥ 0.001 |
| Day +30 (n=58) | 28(48.3%) | 15(25.9%) | 2(3.4%) | 13(22.4%) |
| Day +60 (n=60) | 15(25.0%) | 22(36.7%) | 0(0) | 23(38.3%) |
| Day +90 (n=57) | 12(21.1%) | 14(24.6%) | 1(1.8%) | 30(52.6%) |
| Day +180 (n=50) | 6(12.0%) | 22(44.0%) | 0(0) | 22(44.0%) |
| Day +360 (n=50) | 8(16.0%) | 8(16.0%) | 0(0) | 34(68.0%) |

**Supplemental Table 3. Longitudinal analysis of lymphocyte subset recovery and T-cell polyfunctionality using linear mixed-effects models.**

| **Marker** | **Time**  **coefficient (SE, P)** | **Group(B)**  **coefficient (SE, P)** | **Time×Group(B)**  **interaction (SE, P)** | **Random intercept**  **SD** | **Random slope**  **SD** |
| --- | --- | --- | --- | --- | --- |
| CD3+ (Cells/μl) | 6.142691 (1.437071)  P **<0.001** | 278.8183 (205.7058)  P=0.1764 | -1.060802 (1.627997)  P=0.5152 | 0.9877 | 0.008058 |
| CD4+ (Cells/μl) | 1.297442 (0.437190)  P=**0.0033** | 72.0727 (53.1380)  P=0.1761 | -0.198086 (0.487121)  P=0.6846 | 0.3011 | 0.005501 |
| CD8+ (Cells/μl) | 4.631146 (1.765781)  P=**0.0092** | 222.4985 (153.1294)  P=0.1473 | -0.971805 (2.021087)  P=0.6310 | 1.3077 | 0.019463 |
| NK (Cells/μl) | 1.473966 (0.559636)  P=**0.0089** | 111.2405 (72.6941)  P=0.1271 | -0.709195 (0.632953)  P=0.2635 | 0.8685 | 0.008243 |
| CD19+ (Cells/μl) | 0.865623 (0.232349)  P **<0.001** | 79.2947 (36.1393)  P=**0.0290** | -0.472774 (0.258105)  P=0.0681 | 0.0029 | 0.002457 |
| CD4+ FS | 0.000777 (0.000116)  P **<0.001** | 0.0944 (0.0210)  P **<0.001** | -0.000407 (0.000132)  P=**0.0023** | 0.3144 | 0.001801 |
| CD4+ PFS | 0.000725 (0.000103)  P **<0.001** | 0.0879 (0.0208)  P **<0.001** | -0.000396 (0.000117)  P **<0.001** | 0.4792 | 0.001491 |
| CD8+ FS | 0.000716 (0.000143)  P **<0.001** | 0.1079 (0.0341)  P=**0.0017** | -0.000229 (0.000163)  P=0.1604 | 0.7092 | 0.001231 |
| CD8+ PFS | 0.000656 (0.000132)  P **<0.001** | 0.0945 (0.0307)  P=**0.0023** | -0.000202 (0.000150)  P=0.1813 | 0.6768 | 0.001230 |

Note: Linear mixed-effects models were used to assess longitudinal changes in immune parameters and their associations with aGvHD severity, accounting for repeated measurements within patients. Fixed effects included time (days after transplantation), group, and their interaction, with patient-specific random intercepts and slopes for time. Cohort A included patients with grade II–IV aGvHD, whereas Cohort B included those with no or grade I aGvHD. Group (B) represents Cohort B relative to Cohort A (reference group). Models were fitted using restricted maximum likelihood (REML). A P value < 0.05 was considered statistically significant.

**Supplemental Table 4. Within-person centered correlations between blood counts parameters and T-cell functional reconstitution**

| Laboratory Parameter | T-cell Function Assay | Spearman ρ | 95% CI | P value | P value (Bonferroni) | Pearson r |
| --- | --- | --- | --- | --- | --- | --- |
| WBC (×10⁹/L) | CD4+ FS | 0.44 | (0.34–0.53) | <0.001 | <0.001 | 0.382 |
|  | CD4+ PFS | 0.434 | (0.34–0.53) | <0.001 | <0.001 | 0.365 |
|  | CD8+ FS | 0.41 | (0.31–0.50) | <0.001 | <0.001 | 0.346 |
|  | CD8+ PFS | 0.409 | (0.30–0.50) | <0.001 | <0.001 | 0.341 |
| Lyn (×10⁹/L) | CD4+ FS | 0.423 | (0.33–0.52) | <0.001 | <0.001 | 0.37 |
|  | CD4+ PFS | 0.416 | (0.32–0.51) | <0.001 | <0.001 | 0.351 |
|  | CD8+ FS | 0.394 | (0.29–0.49) | <0.001 | <0.001 | 0.338 |
|  | CD8+ PFS | 0.393 | (0.28–0.49) | <0.001 | <0.001 | 0.332 |

Note: WBC, white blood cell count; Lyn, lymphocyte count; FS, functional score; PFS, polyfunctional score. Within-person deviations calculated as individual observation minus individual mean. Bootstrap 95% CIs based on 2000 resamples. Bonferroni correction applied for 8 simultaneous tests (α = 0.00625).

**Supplemental Table 5. Longitudinal associations of within-person changes in leukocyte and lymphocyte counts with T-cell functional scores using linear mixed-effects models.**

| Laboratory Parameter | T-cell Function assay | Model | β (SE) | P value | P value (Bonferroni) |
| --- | --- | --- | --- | --- | --- |
| WBC (×10⁹/L) | CD4+ FS | Unadjusted | 0.0366 (0.0062) | <0.001 | 3.64e-08 |
|  | CD4+ FS | Adjusted for time | 0.0112 (0.0069) | 0.1063 | 1.0000 |
|  | CD4+ PFS | Unadjusted | 0.0323 (0.0057) | <0.001 | 2.26e-07 |
|  | CD4+ PFS | Adjusted for time | 0.0087 (0.0065) | 0.1796 | 1.0000 |
|  | CD8+ FS | Unadjusted | 0.0431 (0.0081) | <0.001 | 1.48e-06 |
|  | CD8+ FS | Adjusted for time | 0.0127 (0.0093) | 0.1703 | 1.0000 |
|  | CD8+ PFS | Unadjusted | 0.0391 (0.0075) | <0.001 | 2.52e-06 |
|  | CD8+ PFS | Adjusted for time | 0.0106 (0.0086) | 0.2170 | 1.0000 |
| Lyn (×10^10^/L) | CD4+ FS | Unadjusted | 0.0355 (0.0062) | <0.001 | 1.25e-07 |
|  | CD4+ FS | Adjusted for time | 0.0099 (0.0069) | 0.1510 | 1.0000 |
|  | CD4+ PFS | Unadjusted | 0.0311 (0.0058) | <0.001 | 8.59e-07 |
|  | CD4+ PFS | Adjusted for time | 0.0072 (0.0064) | 0.2597 | 1.0000 |
|  | CD8+ FS | Unadjusted | 0.0421 (0.0082) | <0.001 | 3.07e-06 |
|  | CD8+ FS | Adjusted for time | 0.0117 (0.0093) | 0.2057 | 1.0000 |
|  | CD8PFS | Unadjusted | 0.0381 (0.0076) | <0.001 | 5.51e-06 |
|  | CD8+ PFS | Adjusted for time | 0.0095 (0.0086) | 0.2652 | 1.0000 |

Note: All models include random intercept for patient ID. β represents the change in immune function assay per 1-SD increase in within-person deviation of the laboratory parameter. "Adjusted for time" model additionally adjusts for post-transplant day as a continuous covariate. After adjusting for time trend, none of the associations remained statistically significant, suggesting that the observed correlations are largely driven by concurrent time-related recovery trajectories. WBC, white blood cell; Lyn, lymphocyte count; FS, functional score; PFS, polyfunctional score.

**Supplemental Table 6. Univariable logistic regression analysis of factors associated with cGvHD at Day +180.**

| **Variables** | **OR** | **95%CI** | ***p-*value** |
| --- | --- | --- | --- |
| CD3+ cells (%) | 1.4783 | 0.7897-2.7673 | 0.2217 |
| CD4+ cells (%) | 0.5173 | 0.3192-0.8384 | 0.0075 |
| CD8+ cells (%) | 1.9387 | 1.1975-3.1387 | 0.0071 |
| NK cells (%) | 0.7426 | 0.3620-1.5233 | 0.4168 |
| CD19+ cells (%) | 0.5845 | 0.3423-0.9979 | 0.0491 |
| CD3+ cells (/μl) | 1.1730 | 0.4397-3.1294 | 0.7499 |
| CD4+ cells (/μl) | 0.7154 | 0.3634-1.4085 | 0.3326 |
| CD8+ cells (/μl) | 1.0504 | 0.1795-6.1482 | 0.9565 |
| NK cells (/μl) | 0.8246 | 0.3378-2.0130 | 0.6718 |
| CD19+ cells (/μl) | 1.1378 | 0.3822-3.3874 | 0.8166 |
| CD4+ FS | 1.2057 | 0.4872-2.9843 | 0.6858 |
| CD4+ PFS | 1.2066 | 0.4883-2.9814 | 0.6841 |
| CD8+ FS | 1.9510 | 1.2079-3.1514 | 0.0063 |
| CD8+ PFS | 1.8299 | 1.1052-3.0299 | 0.0188 |
| aGvHD (Grade II-IV; Ref: Neg&Grade I) | 0.7406 | 0.3622-1.5145 | 0.4107 |
| Peak CMV-DNA load (IU/mL) | 0.6644 | 0.3599-1.2265 | 0.1911 |
| Bacteremia (< day +100)(Yes; Ref: No) | 0.2936 | 0.2061-0.4184 | <0.001 |

**Supplemental Table 7. Univariable logistic regression analysis of factors associated with cGvHD at Day +360.**

| **Variables** | **OR** | **95%CI** | ***p-*value** |
| --- | --- | --- | --- |
| CD3+ cells (%) | 0.7466 | 0.3615-1.5418 | 0.4296 |
| CD4+ cells (%) | 0.9210 | 0.2349-3.6104 | 0.9210 |
| CD8+ cells (%) | 1.8701 | 1.1395-3.0693 | 0.0133 |
| NK cells (%) | 0.6308 | 0.3541-1.1239 | 0.1179 |
| CD19+ cells (%) | 0.4406 | 0.2857-0.6793 | 0.0002 |
| CD3+ cells (/μl) | 1.0860 | 0.2775-4.2507 | 0.9056 |
| CD4+ cells (/μl) | 0.5967 | 0.3458-1.0297 | 0.0636 |
| CD8+ cells (/μl) | 1.0475 | 0.1698-6.4609 | 0.9601 |
| NK cells (/μl) | 1.4235 | 0.7360-2.7533 | 0.2941 |
| CD19+ cells (/μl) | 0.6583 | 0.3590-1.2070 | 0.1765 |
| CD4+ FS | 1.1585 | 0.4170-3.2189 | 0.7778 |
| CD4+ PFS | 1.1311 | 0.3702-3.4562 | 0.8289 |
| CD8+ FS | 0.9433 | 0.1863-4.7761 | 0.9437 |
| CD8+ PFS | 0.9084 | 0.2564-3.2180 | 0.8817 |
| aGvHD (Grade II-IV; Ref: Neg&Grade I) | 1.6861 | 0.9803-2.9001 | 0.0590 |
| Peak CMV-DNA load (IU/mL) | 0.6375 | 0.3554-1.1435 | 0.1310 |
| Bacteremia (< day +100) (Yes; Ref: No) | 0.5089 | 0.3159-0.8199 | 0.0055 |

**Supplementary Figure 1. Forest plots of multivariable logistic regression analyses of factors associated with CMV reactivation and aGvHD.** **(A)** Factors associated with CMV reactivation. The model included aGvHD, sex, age, primary disease, donor type, ABO compatibility, HCT-CI, R-DRI, CD34+ cell dose, conditioning intensity, time to neutrophil and platelet engraftment, and letermovir prophylaxis. **(B)** Factors associated with aGvHD. The model included the same covariates as in Panel A, with log10-transformed CMV DNA viral load replacing aGvHD. Points represent odds ratios (ORs), and horizontal lines indicate 95% confidence intervals (CIs). The vertical dashed line at OR=1 indicates no association; values above or below 1 indicate higher or lower odds of the outcome, respectively. Statistical significance was defined as P < 0.05. ALL, acute lymphoblastic leukemia; AML, acute myeloid leukemia; CMV, cytomegalovirus; HCT-CI, hematopoietic cell transplantation-specific comorbidity index; R-DRI, refined disease risk index; LMV, letermovir; RIC, reduced-intensity conditioning; MAC, myeloablative conditioning.

**Supplemental Figure 2. Reconstitution of total T cells, CD4^+^ T cells, CD8^+^ T cells , B cells, and NK cells after HSCT.** (A) The counts of different lymphocyte subsets. (B) The proportion of different lymphocyte subsets. (C) The ratio of CD4+/CD8+. Differences across sampling time points were analyzed using the one-way ANOVA analysis. Bar graphs represent with median and interquartile range (IQR), and statistically significant P values are shown. * P < 0.05; ** P < 0.01; *** P< 0.001 and **** P <0.0001 in pairwise comparisons among the groups

**Supplementary Figure 3. Flow cytometry gating strategy for assessing T-cell functional responses.** Representative flow cytometry plots of peripheral blood mononuclear cells (PBMCs) stimulated with staphylococcal enterotoxin B (SEB) or treated with dimethyl sulfoxide (DMSO) as an unstimulated control. Time gating was first applied to exclude acquisition irregularities, followed by forward scatter (FSC) and side scatter (SSC) gating to identify mononuclear cells. Dead cells and CD14+ monocytes were excluded using fixable viability dye (FV) and CD14 staining. Viable CD14-negative cells were then gated into CD4+ (red) and CD8+ (blue) T-cell populations based on CD4 and CD8 expression. Intracellular cytokine staining was used to assess IFN-γ, TNF-α, MIP-1β, and IL-2 production in each population. Representative two-parameter cytokine plots from the DMSO control and SEB-stimulated samples are shown. Fluorescence compensation was applied to minimize spectral spillover between channels.

**Supplemental Figure 4. Longitudinal reconstitution of lymphocyte subsets and T-cell polyfunctionality after allogeneic transplantation. (A-I)** Serial measurements of CD3+, CD4+ and CD8+ T cells, NK cells and CD19+ B cells (absolute counts, cells/μL; A-E) and of CD4+/CD8+ T-cell polyfunctionality (F-I) at days +30, +60, +90, +180 and +360 after transplantation. Thin lines with 30% opacity represent individual patients; bold lines with filled circles represent the mean of each cohort. Red, Cohort A (Grade II–IV aGvHD, n=19); blue, Cohort B (no or Grade I aGvHD, n=57). For the logarithmic panels, values below 1 cell/μL (including values of 0) are plotted at the lower limit of the axis. Numbers beneath each time point indicate the number of patients with available data in Cohort A/ Cohort B. Time points are plotted at equal spacing; no interpolation was performed for missing visits, and lines are interrupted where a visit was not available. FS, functional score; PFS, polyfunctional score.

**Supplemental Figure 5. The interactive effects of aGvHD and CMV reactivation on** **immune reconstitution in HSCT patients.** Two-way ANOVA analysis examine the interaction effects of aGvHD (grade II–IV vs. negative-grade I) and CMV-DNA load (>500 IU/mL vs. <500 IU/mL) on the counts of different lymphocyte subsets and the COMPASS scores.
